# Hormonal Approach for Postmenopausal Vulvovaginal Atrophy

**DOI:** 10.3389/frph.2021.783247

**Published:** 2021-11-29

**Authors:** Ana Paula Ferreira Costa, Ayane Cristine Alves Sarmento, Pedro Vieira-Baptista, José Eleutério, Ricardo Ney Cobucci, Ana Katherine Gonçalves

**Affiliations:** ^1^Health Sciences Postgraduate Program, Federal University of Rio Grande do Norte (UFRN), Natal, Brazil; ^2^Lower Genital Tract Unit, Centro Hospitalar de São João, Porto, Portugal; ^3^Hospital Lusíadas Porto, Porto, Portugal; ^4^Department of Obstetrics and Gynecology, Federal University of Ceará, Fortaleza, Brazil; ^5^Graduate Program of Biotechnology, Potiguar University (UnP), Natal, Brazil; ^6^Department of Obstetrics and Gynaecology, Federal University of Rio Grande do Norte (UFRN), Natal, Brazil

**Keywords:** menopause, vulvovaginal atrophy, hormone therapy, estrogen, DHEA, testosterone, oxytocin

## Abstract

Menopause is a physiological and progressive phenomenon secondary to decreased ovarian follicular reserve that significantly affects the genital tract. Although postmenopausal vulvovaginal atrophy primarily affects postmenopausal women, it is also seen in premenopausal women. The hypoestrogenic condition results in hormonal and anatomical changes, with the main symptoms, are dryness, burning and genital irritation, decreased lubrication, urinary urgency, dysuria, and recurrent urinary tract infections. This review aims to update hormone therapy for urogenital atrophy, both local and systemic, and discusses the importance of understanding and the need for active treatment of this condition. The main therapeutic objective is the relief of symptoms, and hormonal therapy (HT) is still the most effective choice for treating clinical manifestations, despite the side effects of its use. HT should be used in an individualized way to the needs of the women and appropriate to the stage in which she is menopausal, perimenopausal, or after menopause.

## Introduction

Menopause is a gradual and physiological event due to ovarian failure and consequent hypoestrogenism, which significantly affects the lower genital tract. Several denominations are used to describe the alterations noticed in the lower genital tract, such as atrophic vaginitis, vulvovaginal atrophy (VVA), and, lately, genitourinary syndrome of menopause (GSM) (1–3).

Vulvovaginal atrophy of any degree is present in 15% of premenopausal women and in 40–54% of postmenopausal women (4, 5). Women may experience some or all signs and symptoms (vaginal dryness, dyspareunia, sensations of discomfort, burning and irritation, vulvovaginal pruritus, dysuria, and increased frequency of genitourinary infections). The condition is often underdiagnosed due to sexual embarrassment or general disregard associated with it as a liability of natural aging, especially if the symptoms are mild (4, 5).

The main therapeutic objective is the relief of symptoms. Local (vaginal) hormones are the most used option to fulfill this objective. They promote the renovation of the epithelium and vaginal flora, and improvement of the urogenital and sexual complaints (3, 5).

Several hormonal treatment options are currently available, including systemic hormonal therapies (oral and transdermal), low-dose vaginal estrogen, prasterona therapies (2, 3, 5), and Selective Estrogen Receptor Modulators (SERMs) as Ospemifene. This article aims to contribute to knowledge about subjective and objective measurements available for assessing vulvar, vaginal, and lower urinary tract atrophy, as well as future possibilities; so, the hormonal treatment options have been available over the years for this syndrome.

## Systemic

Systemic estrogen therapy is in preference if vasomotor symptoms are also present, whereas local vaginal estrogen therapy (vaginal estrogen ovules or tablets, creams, or a vaginal ring) is preferred when genitourinary symptoms are the only complaint (3–8). Patients receiving systemic ET for other menopausal symptoms frequently have persistent urogenital symptoms requiring supplemental vaginal ET.

Exogenous estrogen use restores normal the vaginal pH, increases epithelial thickness, and revascularizes the epithelium, thus increasing vaginal lubrication. As a result, systemic hormonal therapy relieves symptoms of VVA, including dryness, irritation, pruritus, dyspareunia, and urinary urgency, and may also decrease the incidence of lower urinary tract infections, thus improving the quality of life of these women (5, 9, 10).

In some cases, systemic estrogen therapy is not effective. One of the reasons for this is the fact that, within the intestine, estrogen levels are controlled by a group of intestinal bacteria called estrobolome. When there is an imbalance in the intestine, estrobolome releases an excess enzyme, beta-glucuronidase, which causes harmful estrogens to continually circulate through our system. Depending on the diversity and amount of intestinal bacteria, we may have a low beta-glucuronidase (insufficient reabsorption and a deficit of estrogen in the body) or a high beta-glucuronidase (excess of estrogen being reabsorbed) (11).

## Progesterone

Progesterone is a modulator of normal reproductive functions. These functions include ovulation, uterine and mammary gland development, and neurobehavioral expression associated with sexual responsiveness. Oral micronized progesterone is molecularly identical to human progesterone and provides additional therapeutic benefits for sleep and endometrial hyperplasia protection. However, there is reason to believe that progesterone has a more favorable safety profile than medroxyprogesterone; large safety trials of progesterone as postmenopausal monotherapy are lacking (12–14).

## Testosterone

In postmenopause, there is a decline in the production of androgens, which are also responsible, among others, for sexual function; the administration of testosterone for the treatment of symptoms related to menopause has been studied, with an emphasis on decreasing libido and sexual desire (15).

Testosterone use in postmenopausal women is considered effective in improving sexual function. However, it is a necessary note that each result has its limitations, mainly related to personal and cultural factors, as the studies evaluated women of diversity. Oral testosterone is less used as it undergoes hepatic passage, and there is a decrease in the half-life of the drug, thus needing to be ingested several times a day (three to four times) to maintain the serum level. In addition, oral 17-alpha-alkylated forms of testosterone can cause hepatotoxicity, even at physiological levels, thus increasing the risk of hepatitis, cholestasis, and benign and malignant liver neoplasms (16). It is known that the plasma concentration of total testosterone observed in women in their 40's represents half of that seen in their 20's. However, it should be noted that there is no well-defined biochemical criterion to characterize an androgenic insufficiency since it was not possible to directly correlate the plasma levels of total and free testosterone with the complaints of the patients, reinforcing the need to dose them (17).

## Local (Vaginal)

Low-dose vaginal ET is the most used pharmacological treatment for VVA, being also the most effective and safer option. During the low-dose vaginal ET use, systemic estrogen absorption is minimal, and serum estradiol remains within postmenopausal levels (7–14).

The application of vaginal ET must be adjusted according to tolerability of each woman. However, ET usually starts with nightly application between 2 and 3 weeks and decreases to two to three times per week. The posology of formulations of vaginal ET is evidenced in Table 1. In general, women prefer an improvement of symptoms within a few weeks of initiating treatment. However, many women require 8 to 12 weeks to relieve symptoms. The use of a progestogen is not required for endometrial protection in women receiving low-dose vaginal ET. Nonetheless, periodic endometrial surveillance or progestogen use can be considered for women at high-risk endometrial neoplasia (7–14).

**Table 1:** Hormonal therapy options.

**Treatment**	**Posology**	**Maintenance**
**Vaginal cream:**		
- Estradiol-17b	0.5–1 g day for 2 weeks	0.5 g/day twice a week
- Estriol	0.5 mg day for 2 weeks	0.5 mg/day twice a week
- Conjugated estrogens	0.5–1 g day for 2 weeks	0.5 g/day twice a week
- Promestriene	10 g day for 2 weeks	10 g/day twice a weeks
- Testosterone	2 mg per night 2 weeks	2 mg per night twice a week
**Vaginal tablets**		
- Estradiol hemihydrate	10 μg day for 2 weeks	10 μg/ day twice a week
**Vaginal capsules**		
- Estradiol-17b softgel capsules	4/ 10 /25 μg day for 2 weeks	4 μg/day twice a week
- Capsules DHEA^*^	6.5 mg day for 2 weeks	6.5 mg/day twice a week
**Vaginal ring**		
- Estradiol-17b	2 mg (releases 7.5 μg day for 90 days	2 mg/day
- Estradiol acetate	12.4/24.8 mg (releases 0.05/0.1 μg day)	12.4 mg/day
**Transdermical estrogens**		
- Estradio (gel)	0.5–2.5 g day for 24 a 48 days	0.5 g day
- Testosterone (gel)	50 mg	50 mg
**Oral estrogens**		
- Estradiol	1 mg/day	1 mg/day
- Estriol	1–2 mg/day	1 mg/day
**Others hormonal treatment**		
- Tibolone	2.5 mgorally per day	1.25–2.5 mg orally per day
- Oxytocin gel (intravaginal)	400 IU^**^ per night	400 IU^**^ per night

* *DHEA, Dehydroepiandrosterone*;

** *IU, International unit. Oral testosterone is not recommended for women due to its harmful effects on the lipid profile*.

According to a systematic review conducted by Biehl et al. (18), use of vaginal ET for 1 year was associated with complications, such as vulvovaginal mycosis, vaginal bleeding, endometrial hyperplasia, and endometrial cancer. Another complication that usually not requires treatment is the development of mild transient candidiasis (19). Vaginal ET does not seem to increase the risk of venous thromboembolism, but data on high-risk patients are lacking (7–14). Essentially, non-hormonal treatments are preferred initial strategies for the survivors of hormone-dependent cancers (breast and endometrial cancers). However, after a detailed evaluation of benefits and risks, low-dose vaginal ET, for a short time, could be considered in women with unmanageable symptoms affecting their quality of life (7, 14). Vaginal ET use was not associated with significant absorption, which may provide indirect evidence of safety. Research confirmed this information when observed that vaginal ET is considerable safety in women with breast cancer, receiving aromatase inhibitors (20). Another clinical trial evaluated the efficacy and safety of an ultra-low dose of vaginal gel estriol (0.005%) and demonstrated that the treatment group displayed improved VMI, pH, dryness, global symptoms, and exploratory signs as FSFI scores. Furthermore, serum estrogen, LH, and FSH levels remained unchanged (21). Multiple vaginal estrogen products with similar efficacy are available (Table 1).

## Intravaginal Dehydroepiandrosterone (DHEA)

Dehydroepiandrosterone is a steroid produced in the adrenal glands and converted into sex hormones (estrogens and androgens). A represents an inactive precursor, being transformed into active androgens, only in peripheral tissues that contain the enzymes necessary to continue the steroidogenesis process; thus, each tissue builds its own hormonal identity (intracrinology), avoiding unnecessary exposure to circulating active steroids (22, 23). The vaginal tissue-specific enzymes transform DHEA into the appropriate small amounts of estrogens and androgens for a strictly intracellular and local action, improving the vaginal pH and vaginal maturation index. Furthermore, improvement in sexual arousal and libido has also been reported. The only side effect reported was vaginal discharge due to the melting of the insert (23).

Clinical trial realized using DHEA showed that vaginal prasterone different doses (3.25 and 6.5 mg/day) increased in blood DHEA levels in a dose-dependent manner while serum estradiol was increased only in those insignificant prasterone concentrations. Despite an increase in the level, it is important to cite that the sex steroid levels remained within the lowest levels for postmenopausal women. More studies are necessary to clarify the long-term effects of this treatment (24) (Table 1).

## Testosterone

Although there are few studies about its efficacy, intravaginal testosterone has been used as hormonal therapy that has shown positive effects in relieving vaginal atrophy symptoms and decreased libido. Some research showed that a single intravaginal dose of 2 mg in premenopausal women resulted in supra physiologic testosterone levels with no change in serum estradiol; another study appointed that these hormone treatments reduced the vaginal pH and increased the vaginal cell score and the number of lactobacilli. Finally, a meta-analysis concluded that the effect of vaginal testosterone on sexual function and sexuality scores was similar to estrogen therapy. Thus, larger studies are needed to assess safety and efficacy (25–28) (Table 1).

## Synthetic Steroids and Oxytocin

Tibolone, a synthetic steroid, is rapidly converted to three active metabolites (3α-OH, 3β-OH, and 1Δ4), all of which are biologically active and contribute to the hormonal profile of the parent compound. It has been used in postmenopausal women to relieve vasomotor symptoms and improve vaginal atrophy, changing the vaginal maturation index and increasing the sex drive through its part androgenic properties. Moreover, urinary incontinence problems of nocturia and urgency were found to be improved (5, 18, 29, 30) (Table 1).

Oxytocin has been evaluated as an alternative due to concerns about the use of estrogen therapy. It is a peptide hormone produced in the hypothalamus, and it is best known for its role in labor and lactation. Some studies suggest that applying oxytocin produces a healthier and more vaginal epithelium, with normal vaginal pH levels and a significant reduction in symptoms. However, other research is necessary for confirmation of these results (31–35).

## Conclusion

Hormonal therapy must be individualized to the needs of women and is conditioned by the stages of menopause. Low-dose vaginal estrogens, vaginal DHEA, and systemic estrogen therapy are effective for moderate to severe GSM. Treatment by administering hormone systemic, in particular, is commonly used to combat vasomotor symptoms, preserve bone mass, improve sleep, prevent the deterioration of cognitive function, and stimulate libido. The vaginal formulations promote the renovation of the epithelium and vaginal flora and improve the urogenital and sexual complaints, decreasing the vaginal dryness. Tibolone is another option used to relieve vasomotor symptoms and improve vaginal atrophy, changing the VMI and increasing the sex drive through its androgenic properties. Oxytocin has been evaluated as an alternative due to concerns about the use of estrogen therapy. However, other research is necessary for confirmation of these results.

## Author Contributions

AC and AG: conceived and designed the study. AC, AS, AG, and JE: drafted and revised the article where appropriate. AS and AC: prepared the table. AG, PV-B, JE, and RC: carried out the final revision of the manuscript. All authors contributed to the article and approved the submitted version.

## Conflict of Interest

The authors declare that the research was conducted in the absence of any commercial or financial relationships that could be construed as a potential conflict of interest.

## Publisher's Note

All claims expressed in this article are solely those of the authors and do not necessarily represent those of their affiliated organizations, or those of the publisher, the editors and the reviewers. Any product that may be evaluated in this article, or claim that may be made by its manufacturer, is not guaranteed or endorsed by the publisher.
